# Psoriasis and its impact on close relatives and partners of patients – A cross‐sectional questionnaire study

**DOI:** 10.1002/ski2.355

**Published:** 2024-03-15

**Authors:** Katja Grossschaedl, Wolfgang Weger, Thomas Graier, Wolfgang Salmhofer, Ulrich Mrowietz, Peter Wolf

**Affiliations:** ^1^ Department of Dermatology and Venereology Medical University of Graz Graz Austria; ^2^ Psoriasis‐Center at the Department of Dermatology University Medical Center Schleswig‐Holstein Kiel Germany

## Abstract

**Background:**

Little is known about the exact impact of psoriasis on the disease burden of close relatives and partners of those affected by the disease.

**Objectives:**

The aim of this single‐centre cross‐sectional study was to evaluate the quality of life in psoriasis patients and the impact of disease on partners and close relatives.

**Methods:**

250 plaque‐type psoriasis patients (58.4% males and 41.6% females) with mostly treatment‐controlled disease (mean PASI of 1.7 and Dermatology Life Quality Index (DLQI) of 4.1) were recruited from the Psoriasis Registry Austria (PsoRA) and their close relatives and partners were invited to participate in the study. Patient Family Impact Score (PFIS) was calculated from the FamilyPso questionnaire data to establish categories of disease burden in close relatives and partners.

**Results:**

Valid FamilyPso questionnaires were returned from 153 (61.2%) close relatives and partners. Correlation analysis revealed a significant association between PASI and DLQI (*r* = 0.512, *p* < 0.001), PASI and PFIS (*r* = 0.228, *p* = 0.006), and DLQI and PFIS (*r* = 0.210, *p* = 0.014). An at least small or larger impairment of life quality (DLQI ≥ 2) was observed in 46.7% of psoriasis patients, despite treatment. A small or larger disease burden was detected in nearly 78.7% of the male and 77.3% of the female relatives and partners quantified with categorized PFIS.

**Conclusions:**

The study revealed a significant impact of patients' psoriasis on the disease burden of close relatives and partners, depending on the severity of PASI and extent of quality of life disruption in patients. The gender of the relatives and partners had no impact on the PFIS.

## Graphical abstract

This cross‐sectional questionnaire study was done to evaluate the impact of psoriasis disease on close relatives and partners. The study revealed a significant impact of patients psoraisis on the disease burden of close relatives and partners, depending on the severity of PASI and extent of quality of life disruption in patients. The gender of the social environment had no impact on the disease burden. The graphical abstract shows the categories of impairment by Patient Family Impact Score.

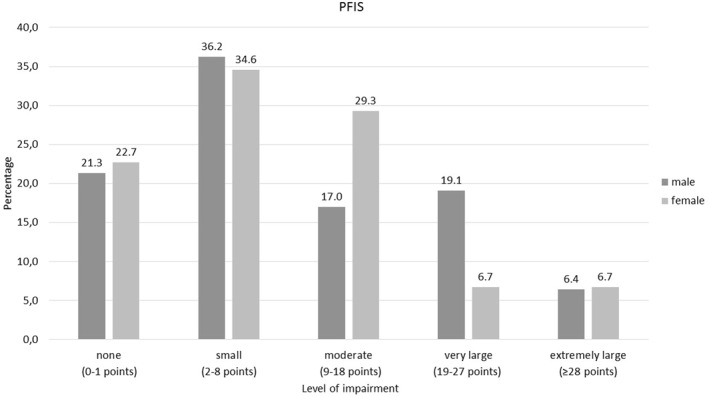

**What's already known about this topic?**

- Previous studies have shown that psoriatic the disease‐burden has an impact of close relatives and partners of those affected by the disease.

**What does this study add?**

- The study revealed a significant impact of patients' psoriasis on close relatives and partners, depending on the severity of Psoriasis Area and Severity Index (PASI) and extent of quality of life disruption in patients.
- The categorized impact of disease burden was larger in close relatives and partners than the impairment of quality of life in the patients when disease was mostly well controlled by treatment.
- The gender of the relatives and partners had no impact on the Patient Family Impact Score (PFIS).

## 1 INTRODUCTION

Mental disorders such as depression or anxiety are significantly more frequent among patients with underlying psoriasis as compared to the general population.^1^, ^2^ The disease burden comprises physical and psychological aspects, creating a “vicious circle” in which the visible skin changes, and disfigurements negatively impact the body image. This, in turn, causes social withdrawal and behavioural avoidance and results in stress, which exacerbates and induces the recurrence of psoriasis.^3^ Moreover, mood disorders are suspected of causing an elevated risk of developing other concurrent diseases,^4^ including especially depression as potential result of neuroinflammation with psoriatic metabolic‐inflammatory patterns.^5^ In 2014, the World Health Organization highlighted the psychological burden of this disease based on stigmatisation and inadequate treatment,^6^ showing that this overall burden significantly reduced the quality of life of patients suffering from psoriasis.^7^ In fact, Rapp et al.^8^ showed an even higher impairment of health‐related quality of life in psoriasis patients as compared to patients suffering from internal chronic diseases, such as myocardial infarction, congestive heart failure and cancer. Stigmatisation is common in psoriasis and prejudices often occur against patients with visible skin changes like psoriasis.^9^ For instance, Sommer et al. demonstrated that the majority of participants of telephone surveys did say that they did not want to get in touch with psoriasis patients. Thus, interventions reducing stigmatisation are necessary.^9^ Weinberger et al. reported about effectiveness of structured short intervention against stigmatisation by measures such as self‐reflection, education, and contact between people and psoriasis patients.^10^

Specifically designed questionnaires can be used to reliably assess the quality of life of psoriasis patients by measuring social, physical and psychological aspects of their lives. These tools provide fundamental assistance and important information about the patients' potential therapeutic adherence.^7^ The social environment and especially psychosomatic stress events play crucial roles in disease progression caused by emotional disturbance.^11^ Distressing events involving family members can increase mental disorders such as anxiety and depression in psoriatic patients.^12^ Several circumstances such as limitations in taking part in social activities, taking vacations and experiencing difficulties in performing everyday life activities diminish the quality of life of affected patients.^11^ These limitations also impact the close relatives and partners of these patients.^11^, ^13^ In general, many studies demonstrate the psychosomatic burden of psoriasis in affected patients; however, data on the impact of this chronic inflammatory disease on close relatives and partners is limited. Therefore, in this study, we aimed to evaluate the impact of psoriasis on close relatives and partners.

## 2 MATERIAL AND METHODS

### 2.1 Study design

This study was conducted as a monocentric, horizontal, single‐centre, cross‐sectional questionnaire study to measure the impact of psoriasis on close relatives and partners. The study was performed between September and December 2021 and involved 250 chronic plaque‐type psoriasis patients who presented at the outpatient clinic of the Department of Dermatology (Medical University of Graz, Austria) and their close relatives and partners. Patients aged 18 and above who had been diagnosed with chronic plaque psoriasis and their relatives and partners were eligible to participate. Data on patient demographics, disease and treatment characteristics, as well as on their comorbidities and Dermatology Life Quality Index (DLQI) were collected and entered into the electronic data bank of the Psoriasis Registry Austria (PsoRA).^14^, ^15^, ^16^ Self‐reported plaque severity and the clinical calculation of PASI were used to determine disease severity. In order to collect detailed information about close relatives and partners, every enrolled patient was provided with a demographic partner report form and the FamilyPso questionnaire^17^ and asked to ask their close partner or relative to participate in the study. A prepaid envelope was provided, enabling the patient to return the demographic partner report form and FamilyPso questionnaire to the study centre free of charge. This study was conducted in accordance with the Declaration of Helsinki and with the approval of the Medical University of Graz Ethics Committee (Ethic no. 33‐413 ex 20/21). FamilyPso questionnaires that were returned by the first 50 partners or relatives together with certain patient data were transmitted according to the study protocol to Ulrich Mrowietz (Psoriasis‐Center at the Department of Dermatology, University Medical Center Schleswig‐Holstein, Kiel, Germany), the principal investigator of the ongoing International FamilyPso study, for comparative analysis. All participating patients gave their written informed consent.

### 2.2 Questionnaires

To quantify the disease‐related impairment of life quality, the German version of the DLQI was used.^18^ The validated FamilyPso questionnaire^17^ was used to determine the burden placed on partners or close relatives of psoriasis patients. This questionnaire consists of 15 items and can be specified into three subgroups (emotional, leisure and social domain). Three recapitulatory domains (emotional, social and leisure domains) can be categorised by using this self‐assessment scale; the lower FamilyPso scores indicate a lower burden on the social environment.^17^ Each item spreads over a 5‐point Likert scale (0 = not true, 1 = somewhat true, 2 = moderately true, 3 = quite true, 4 = very true, or does not apply to me). Cut‐off values for a normal‐to‐moderate (75 percentile) and a very high (90 percentile) psoriasis‐related burden on close relatives or family members were described.^17^ In order to generate a categorised disease burden on relatives and partners of psoriasis patients, points of answers on the 15 items of the FamilyPso questionnaire were summarised to establish a PFIS with a theoretical maximum of 60. Taking into consideration the results of Mrowietz et al.,^17^ the categories of burden were defined as follows: 0–1, none; small, ≥2– <9; moderate, ≥9– <19; very large; ≥19– <28; extremely large, ≥28.

### 2.3 Statistical analysis

The answer “not relatable to me” in the items of the FamilyPso was considered to be “not true”, as intended. However, this study also evaluated non‐relatable items as missing values, consequently reducing the total number of available items (adjusted FamilyPso). The Spearman correlation coefficient was used to evaluate correlations among PASI, DLQI and PFIS. Students *t*‐test or Mann‐Whitney *U* test were performed to analyse differences in the DLQI and FamilyPso or adjusted FamilyPso regarding the patient's gender, the gender of the partners or relatives, concomitant arthritis or depression, body‐site involvement (scalp, palmar and/or plantar, genital/inverse and nail involvement) and administered treatment. The analysis of variances or Kruskal‐Wallis test with a Bonferroni post‐hoc correction was used to compare DLQI and FamilyPso in PASI severity groups (complete remission, PASI > 0 but ≤3, PASI > 3 but ≤10, and PASI > 10). Pearson Chi Square test was used to analyse distribution of comorbidities between participating patients and patients, whose partners/relatives responded to FamilyPso. However, FamilyPso was also calculated by excluding non‐relatable items and adjusting the dividend (adjusted FamilyPso). Statistical analyses were performed using SPSS V27.0 (IBM Corp. Armon, NY). Graphics were designed with Microsoft Office 365 (Microsoft Corporation, Redmond, USA). Statistical significance was set at *p* < 0.05.

## 3 RESULTS

### 3.1 Patient characteristics

Two hundred and fifty psoriasis patients (146 men, 104 women) were enrolled in this study who had a mean (SD) age of 47.95 (14.9) years. The mean (SD) disease duration was 19.41 (14.3) years, and the mean (SD) Body mass index was 28.41 (5.6) (Table 1). Psoriatic arthritis was the most frequently patient self‐reported concomitant disease (45.6%), followed by hypertension (27.6%), hyperlipidaemia (11.6%), depression (9.6%), diabetes (5.6%), coronary heart disease (5.2%) and inflammatory bowel disease (1.6%). Smoking was reported by 38.4% of patients (Table 2). In general, participating patients had a low severity of psoriasis as measured by a mean (SD) PASI of 1.66 (3.6). The mean (SD) DLQI was 4.09 (6.2) (Table 1). Patient characteristics of the overall population and those patients whose close relatives or partners provided FamilyPso responses are summarised in Table 1.

**TABLE 1:** Patient characteristics.

Characteristic			Overall population	FamilyPso responders
Total no. of patients (%)			250 (100)	153 (61.2)
	Male		146 (58.4)	88 (57.5)
	Female		104 (41.6)	65 (42.5)
Mean age (SD)			47.95 (14.9)	49.79 (13.9)
Mean BMI (SD)			28.41 (5.6)	28.79 (5.4)
Mean duration of psoriatic disease (years) (SD)			19.41 (14.3)	20.91 (14.1)
Mean PASI (SD)^$^			1.66 (3.6)	1.59 (2.9)
Mean DLQI (SD)			4.09 (6.2)	3.63 (5.2)
No. (%) of prescribed treatments	Topical therapeutics (only)		41 (16.4)	25 (16.3)
	Oral drugs	All together	17 (6.8)	9 (5.9)
		Apremilast	10 (58.8)	5 (55.)
		Fumaric acid esters	1 (5.9)	1 (11.1)
		JAK inhibitors	4 (23.5)	2 (22.2)
		Filgotinib	1	1
		Tofacitinib	1	1
		Upadacitinib	2	0
		Methotrexate	2 (11.8)	1 (11.1)
	Biologics	All together	192 (76.8)	119 (77.8)
		TNF‐alpha inhibitors	29 (15.1)	21 (17.6)
		Adalimumab	19 (9.9)	14 (11.8)
		Etanercept	9 (4.7)	6 (5.0)
		Golimumab	1 (0.5)	1 (0.8)
		IL 12/23 inhibitor	51 (26.6)	31 (26.1)
		IL 17 inhibitors (all together)	58 (30.2)	33 (27.7)
		Brodalumab	2 (1.0)	1 (0.8)
		Ixekizumab	41 (21.4)	20 (16.8)
		Secukinumab	15 (7.8)	12 (10.1)
		IL 23 inhibitors (all together)	54 (28.1)	34 (28.6)
		Guselkumab	29 (15.1)	19 (16.0)
		Risankizumab	25 (13.0)	15 (12.6)
Number (percentage of patients) with specific body‐site involvement	Scalp^+^		122 (48.8)	72
	Palmar and or plantar*		35 (14.0)	23
	Inverse/genital^#^		77 (30.8)	44
	Nails^§^		107 (42.8)	65
Number (percentage of patients)	PASI group^$^	CR	92 (38.8)	54 (37.8)
		>0 but ≤3	110 (46.4)	65 (45.5)
		>3 but ≤10	25 (10.5)	19 (13.3)
		>10	10 (4.2)	5 (3.5)

Abbreviations: BMI, Body mass index; DLQI, Dermatology Life Quality Index; IL, Interleukin; JAK, Janus Kinase; PASI, Psoriasis Area and Severity Index; PDE, Phosphodiesterase.

^$^ Unknown for 13 (8.5%) patients; 10 (6.5%) patients participating in FamilyPso.

^+^ Unknown for 9 (3.6%) patients; 2 (1.3%) patients participating in FamilyPso.

^*^ Unknown for 5 (2.0%) patients; 1 (0.7%) patient participating in FamilyPso.

^#^ Unknown for 47 (18.8%) patients; 30 (19.6%) patients participating in FamilyPso.

^§^ Unknown for 12 (4.8%) patients, 3 (2.0%) patients participating in FamilyPso.

**TABLE 2:** Self‐reported comorbidities of patients.

Type of comorbidity	Overall population (*N* = 250) (percentage)	Patients with FamilyPso (*n* = 153) (percentage)
Arthritis	114 (45.6)	71 (46.4)
Hypertension	69 (27.6)	49 (32.0)
Hyperlipidaemia	29 (11.6)	21 (13.7)
Depression	24 (9.6)	17 (11.1)
Diabetes	14 (5.6)	11 (7.2)
Coronary heart disease	13 (5.2)	7 (4.6)
Inflammatory bowel disease	4 (1.6)	1 (0.7)
Smoking cigarettes	97 (38.4)	51 (33.3)

No statistically significant difference was observed between the overall population and the FamilyPso responders (*p* = 0.88, Chi Square test).

### 3.2 Characteristics of relatives and partners

**FIGURE 1:**
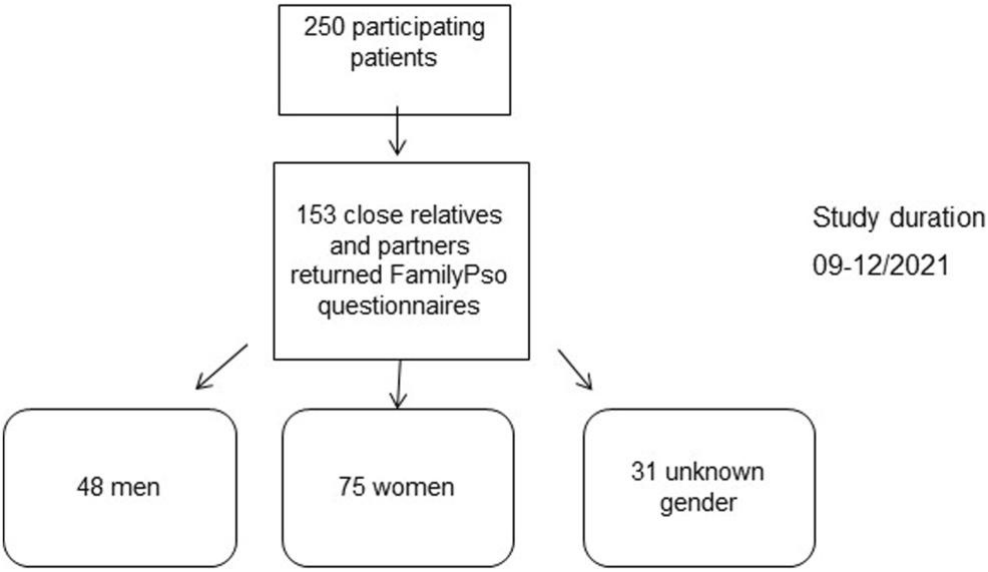
Flow chart of the study design.

Close relatives and partners from 154 psoriasis patients returned the close relative or partner report form and the FamilyPso questionnaire. Data from one close relative had to be excluded due to a missing FamilyPso questionnaire, resulting in total of 153 (61.2%) evaluable FamilyPso questionnaires (Table 1). The majority of responders were females (*n* = 75, 49.0%); males (*n* = 47, 30.7%), and the gender of the relative or partner was unknown in 31 cases (20.3%) (Table 3, Figure 1). Responders were mostly spouses (*n* = 82, 53.6%) or unmarried partners (*n* = 39, 25.5%), and the degree of relationship was unknown in 31 cases (20.3%). In one case (0.6%) the questionnaire was filled out by the patient's mother (Table 3). The age of the participating close relatives and partners ranged from 18 to 82 years.

**TABLE 3:** Characteristics of close relatives and partners.

No. of close relatives and partners (%)		*N* = 153 (100)
Gender	Male	47 (30.7)
	Female	75 (49.0)
	Unknown	31 (20.3)
Relationship status	Married	82 (53.6)
	Husband	30 (19.6)
	Wife	52 (34.0)
	In a relationship	39 (25.5)
	Male	17 (11.1)
	Female	22 (14.4)
	Unknown relationship	31 (20.3)
	Mother	1 (0.6)

### 3.3 Treatment

Biologics were the most frequently administered treatment in this patient cohort (*n* = 192, 76.8%) (Table 1). Seventeen (6.8%) of the patients received oral drugs and 41 (16.4%) patients received topical treatment only. Similar rates were observed for patients whose relatives or partners returned the FamilyPso questionnaire. For detailed information and specific treatments, see Table 1.

### 3.4 Dermatology Life Quality Index

**FIGURE 2:**
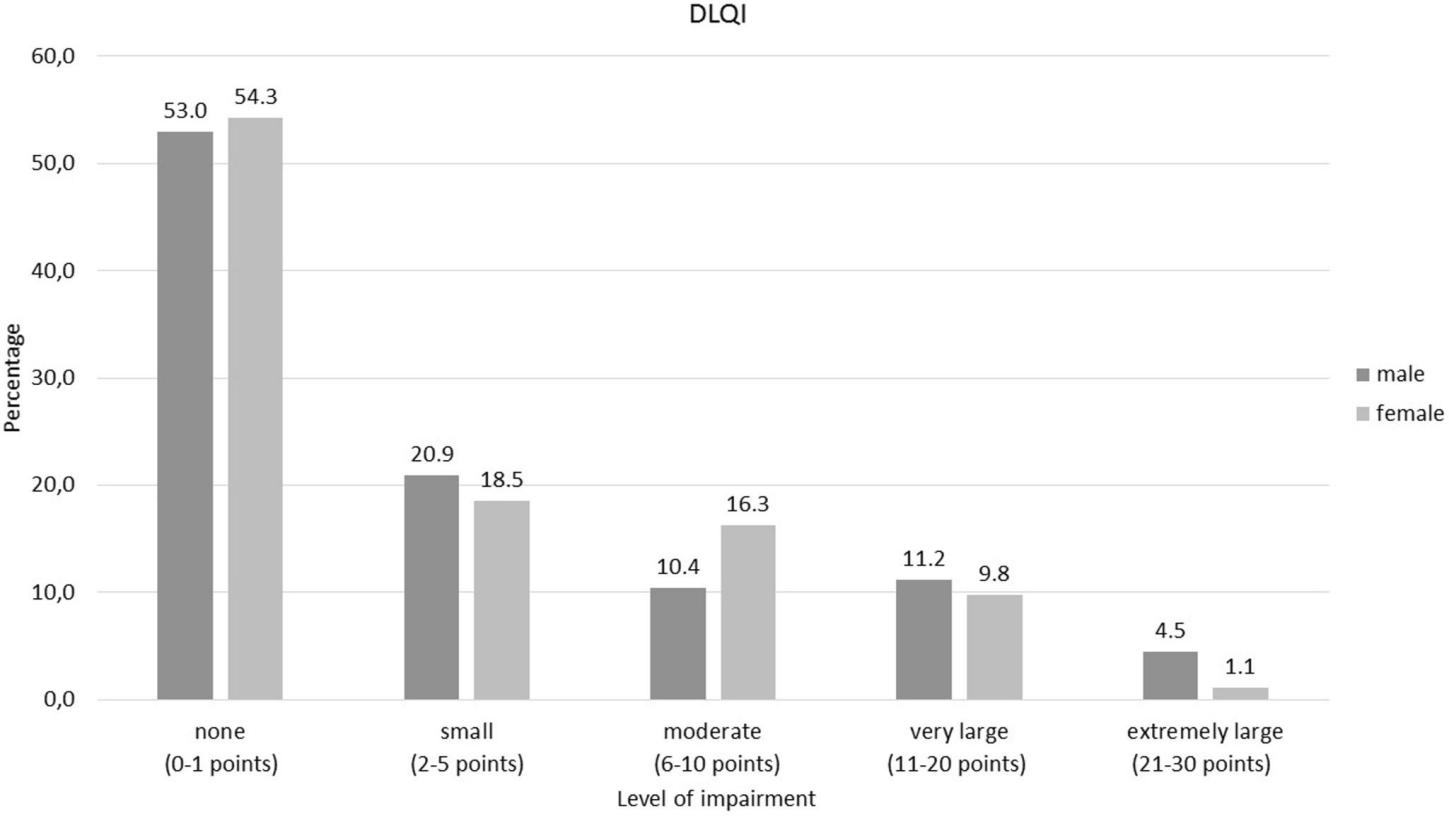
Categories of Impairment in Dermatology Life Quality Index (DLQI). Level of impairment of patients assessed by DLQI divided by gender grouped by points in percentage.

The DLQI form was completed by 226 patients (90.4%). Of these, 121 patients (53.5%) reported no impact on their health‐related quality of life (DLQI: 0–1); 45 patients (19.9%) reported a small impact (DLQI: 2–5); 29 patients (12.9%), a moderate impact (DLQI: 6–10); 24 patients (10.6%), a very large impact (DLQI: 11–20); and 7 patients (3.1%), an extremely large impact on the patient's life (DLQI: 21–30) (Figure 2, Table S1). The results indicate that the health‐related quality of life was not altered by the patient's gender (*p* = 0.335), presence of arthritis (*p* = 0.173) or depression (*p* = 0.942) (Table 4). Furthermore, no statistically significant differences in DLQI were identified regarding the involvement of nails (*p* = 0.54), scalp (*p* = 0.095), or genital/inverse areas (*p* = 0.262). However, a significant difference was observed for patients with palmar and/or plantar involvement with a mean (SD) DLQI of 6.74 (6.40) as compared to 3.60 (5.91) in patients without palmar and/or plantar involvement (*p* = 0.007) (Table 4). Furthermore, patients who received systemic treatments had a significantly lower mean (SD) DLQI of 2.99 (5.11) than patients who received topical treatment, who had a DLQI of 10.58 (7.71) (*p* < 0.001). Specific DLQI results are shown in Table 4. Additionally, significant differences were observed regarding self‐reported plaque severity (*p* < 0.001). Subgroup analysis results revealed a lower DLQI (mean [SD]) in patients with mild plaques (2.29 [3.97]) as compared to moderate plaques (7.86 [6.50], *p* < 0.001) and severe plaques (16.83 [9.91], *p* < 0.001), but not as compared to patients with moderate or severe plaque severity (*p* = 0.170). The DLQI was significantly lower in patients with a complete remission (CR) (1.98 [4.08]) than in patients with PASI‐values > 0 but ≤3 (3.29 [4.26]) (*p* = 0.002), PASI > 3 but ≤10 (9.92 [8.04]) (*p* < 0.001), and PASI > 10 (16.90 [9.49]) (*p* < 0.001). Significant differences were also observed for patients with PASI > 0 but ≤3 and >3 but ≤10 (*p* = 0.002) as well as for patients with PASI > 0 but ≤3 compared to those with PASI >10 (*p* > 0.001) (Table 4). Finally, results of a correlation analysis revealed a significant correlation between PASI and DLQI (*r* = 0.512, *p* < 0.001).

**TABLE 4:** Subgroup analysis of Dermatology Life Quality Index (DLQI) and FamilyPso scores.

Patient characteristic		DLQI, mean (SD)	*p*‐value	FamilyPso score, mean SD)	*p*‐value	Adjusted FamilyPso score, mean (SD)	*p*‐value	PFIS, mean (SD)	*p*‐value
Gender of patient	Female	3.62 (5.18)	0.335	NA	NA	NA	NA	NA	NA
	Male	4.43 (6.75)							
Gender of relative/partner^$^	Female	NA	NA	0.61 (0.69)	0.554	0.67 (0.71)	0.247	9.19 (10.42)	0.554
	Male			0.69 (0.70)		0.83 (0.79)		10.34 (10.47)	
Psoriatic arthritis	Yes	5.08 (7.30)	0.173	0.81 (0.73)	0.099	0.94 (0.78)	0.110	12.20 (10.90)	0.099
	No	3.28 (4.90)		0.61 (0.76)		0.72 (0.86)		9.20 (11.40)	
Concomitant depression	Yes	4.19 (5.74)	0.942	0.84 (0.84)	0.452	0.89 (0.97)	0.707	12.52 (12.60)	0.452
	No	4.09 (6.21)		0.69 (0.74)		0.81 (0.81)		10.35 (11.08)	
Systemic treatment	Yes	2.99 (5.11)	**< 0.001**	0.73 (0.77)	0.430	0.82 (0.82)	0.843	10.91 (11.57)	0.430
	No	10.58 (7.71)		0.60 (0.62)		0.85 (0.88)		8.96 (9.37)	
PASI group	CR	1.98 (4.08)	< **0.001** ^*^	0.53 (0.63)	0.240	0.67 (0.78)	0.640	7.93 (9.45)	0.240
	>0 but ≤3	3.29 (4.26)		0.80 (0.84)		0.85 (0.84)		12.02 (12.61)	
	>3 but ≤10	9.92 (8.04)		0.67 (0.64)		0.80 (0.65)		10.05 (9.65)	
	>10	16.90 (9.49)		0.83 (0.60)		0.90 (0.54)		12.40 (8.96)	
Plaque severity	Mild	2.29 (3.97)	**< 0.001** ^#^	0.69 (0.80)	0.748	0.83 (0.90)	0.804	10.34 (11.95)	0.748
	Moderate	7.86 (6.50)		0.72 (0.62)		0.77 (0.65)		10.84 (9.35)	
	Severe	16.83 (9.61)		0.95 (0.54)		1.02 (0.45)		14.20 (8.07)	
Scalp involvement	Yes	4.92 (7.00)	0.095	0.71 (0.76)	0.983	0.80 (0.81)	0.789	10.60 (11.46)	0.983
	No	3.29 (4.71)		0.70 (0.74)		0.84 (0.85)		10.56 (11.12)	
Nail involvement	Yes	5.36 (7.53)	0.54	0.60 (0.77)	0.175	0.69 (0.79)	0.120	9.00 (11.51)	0.175
	No	3.10 (4.64)		0.77 (0.73)		0.90 (0.85)		11.50 (10.89)	
Palmar and/or plantar involvement	Yes	6.74 (6.40)	**0.007**	0.72 (0.92)	0.922	0.92 (1.14)	0.688	10.87 (13.80)	0.922
	No	3.60 (5.91)		0.71 (0.72)		0.81 (0.86)		10.62 (10.78)	
Inverse and/or genital involvement	Yes	4.96 (6.45)	0.262	0.80 (0.85)	0.169	0.92 (0.79)	0.270	12.00 (11.20)	0.169
	No	3.29 (5.15)		0.62 (0.68)		0.75 (0.82)		9.24 (10.27)	

*Note*: *P*‐values in bold are statistically significant.

Abbreviation: CR, complete remission.

^$^ Data from 31 relatives and partners were excluded due to missing gender report.

^*^ Kruskall‐Wallis test and Bonferroni adjusted subgroup analysis revealed significant differences in DLQI between patients with a CR. and a PASI >0 but ≤3 (*p* = 0.002), a PASI >3 but ≤10 (*p* < 0.001), and a PASI >10 (*p* < 0.001). Significant differences were also observed for patients with a PASI >0 but ≤3 and >3 but ≤10 (*p* = 0.002) and for patients with a PASI >0 but ≤3 and >10 (*p* < 0.001). No significant differences were found between patients with a PASI >3 but ≤10 and >10 (*p* = 1.000).

^#^ Bonferroni‐adjusted subgroup analysis revealed significant differences in DLQI between patients with mild or moderate plaque severity (*p* < 0.001), mild and severe plaque severity (*p* < 0.001), but not between patients with moderate or severe plaque severity (*p* = 0.170).

### 3.5 FamilyPso questionnaire results

**FIGURE 3:**
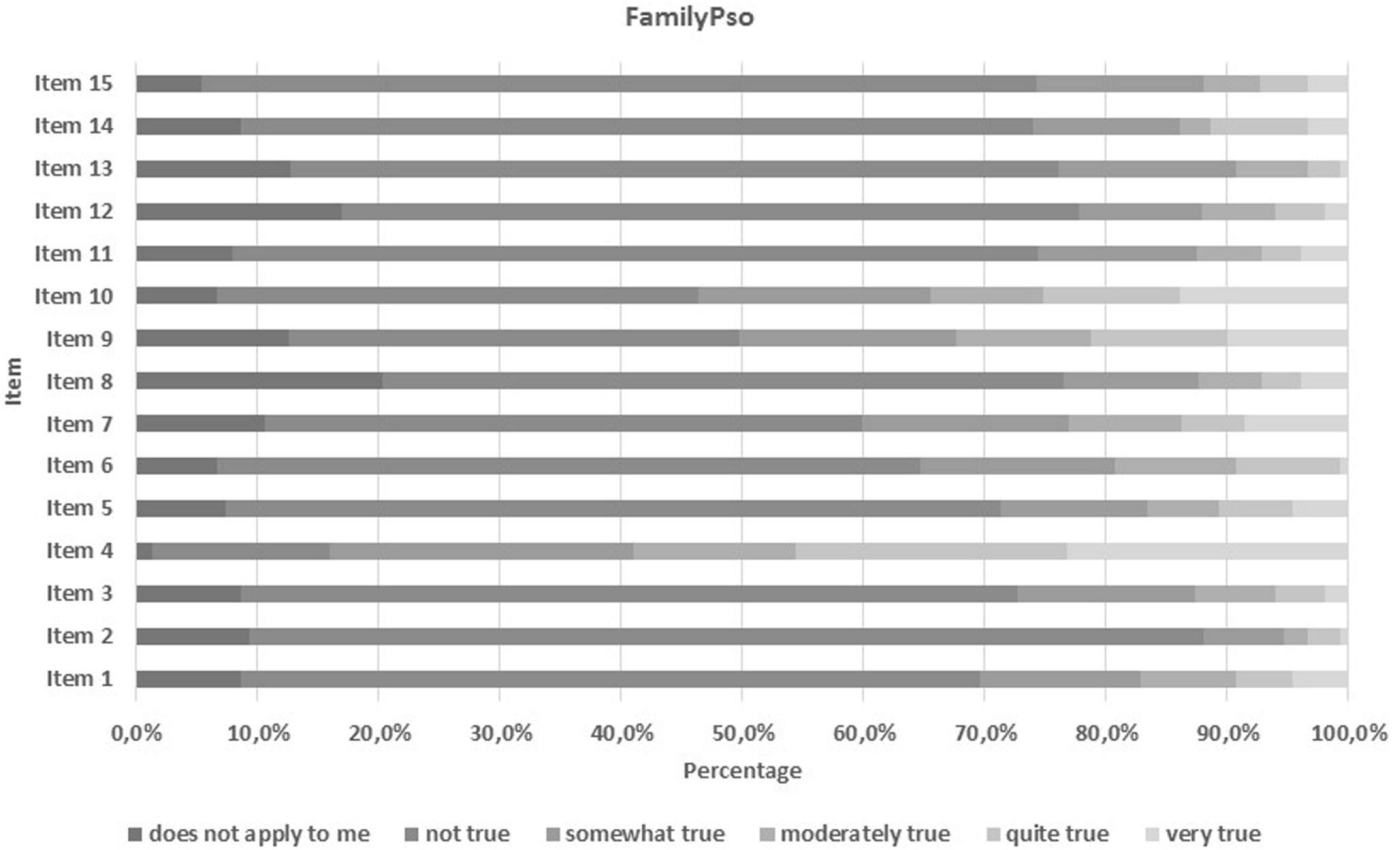
Heat map of the Patient Family Impact Score (PFIS) showing the impact of close relatives and partners for the 15 items.

**FIGURE 4:**
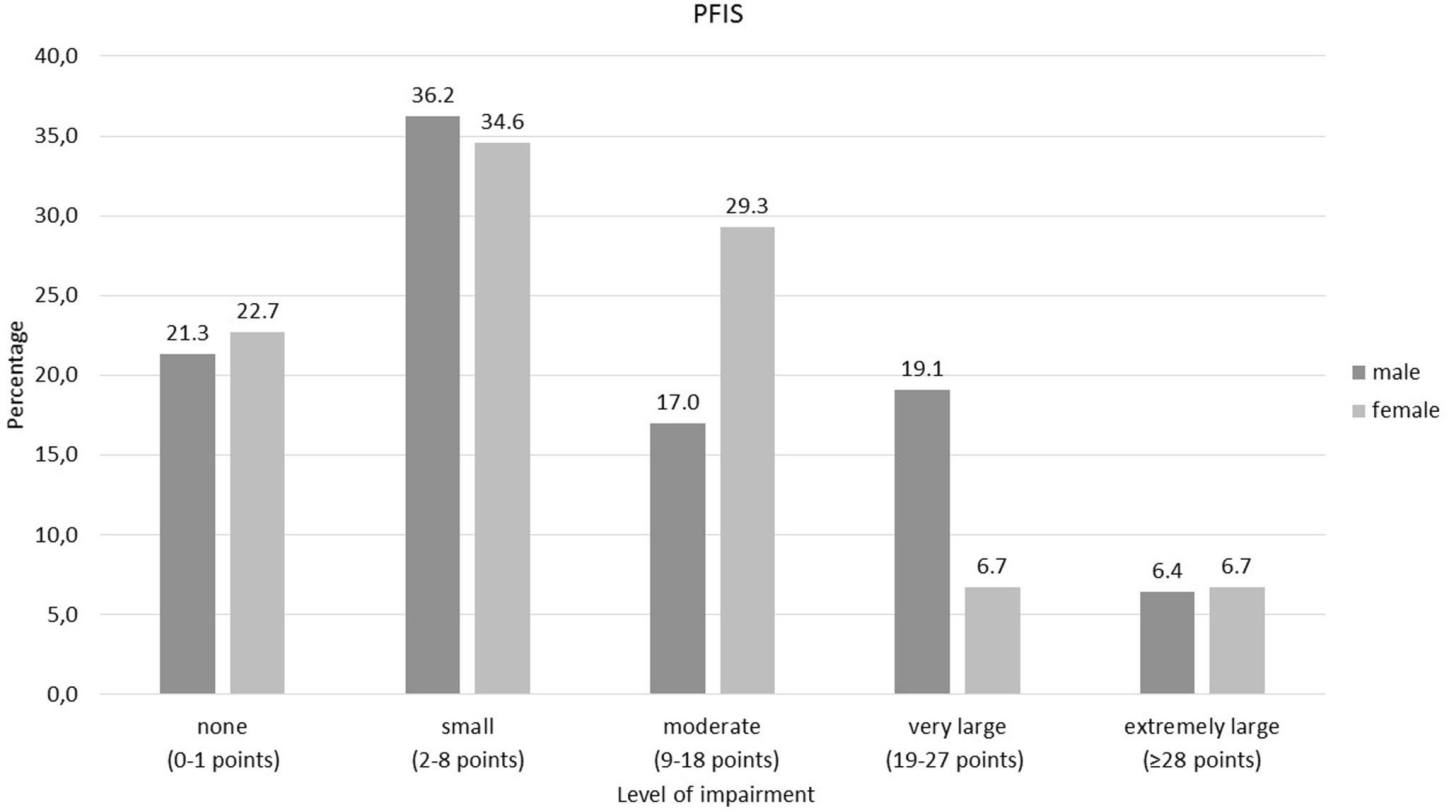
Categories of impairment by Patient Family Impact Score (PFIS). Level of impairment of close relatives and partners assessed by PFIS divided by gender grouped by points in percentage.

The highest psoriasis‐related burden reported by partners or relatives was found for the items in the emotional domain, followed by items in the leisure and social domains (Table 5). The highest scoring items were item 4 (feeling sympathy towards sick partner or relative due to their psoriasis) with a mean (SD) of 2.15 (1.42), item 9 (pain or sleeping problems experienced by relatives or partners because their psoriasis causes stress) with a mean of 1.30 (1.42), and item 10 (relative's/partner's psoriasis outbreaks cause stress) with a mean of 1.36 (1.49) (Table 5, Figure 3). The subgroup analysis revealed no significant differences in FamilyPso items regarding the presence of psoriatic arthritis, concomitant depression, administration of systemic treatment, PASI groups, plaque severity, or an involvement of nails, scalp, palms and/or soles, or genitals (Table 4). No differences in the single‐item analysis were identified regarding the gender of the partners or relatives. The Spearman correlation analysis results revealed a correlation between PASI and PFIS (*r* = 0.228, *p* = 0.006) and between DLQI and PFIS (*r* = 0.210, *p* = 0.014). If we examine the categorised burden of disease in close relatives and partners, we observe a slight to very high burden of disease in overall 78.7% of male and 77.3% of female partners and relatives, as measured by PFIS (Figure 4, Table S2).

**TABLE 5:** FamilyPso items and domains.

FamilyPso item and domains, mean (SD)	All together	Males	Females	*p*‐value
Item 1	0.67 (1.15)	0.69 (0.70)	0.61 (0.69)	0.615
Item 2	0.24 (0.70)	0.63 (0.97)	0.54 (0.99)	0.556
Item 3	0.53 (0.96)	0.50 (0.89)	0.49 (0.99)	0.968
Item 4	2.15 (1.42)	2.27 (1.40)	1.92 (1.43)	0.198
Item 5	0.65 (1.17)	0.74 (1.23)	0.57 (1.15)	0.471
Item 6	0.69 (1.04)	0.63 (1.02)	0.64 (1.01)	0.965
Item 7	0.96 (1.33)	0.67 (1.18)	0.89 (1.26)	0.371
Item 8	0.59 (1.10)	0.42 (0.94)	0.50 (0.97)	0.715
Item 9	1.30 (1.42)	1.53 (1.41)	1.04 (1.34)	0.086
Item 10	1.36 (1.49)	1.51 (1.42)	1.09 (1.40)	0.121
Item 11	0.54 (1.05)	0.68 (1.16)	0.32 (0.75)	0.091
Item 12	0.51 (0.99)	0.55 (0.99)	0.47 (0.95)	0.681
Item 13	0.43 (0.81)	0.47 (0.89)	0.29 (0.62)	0.264
Item 14	0.60 (1.13)	0.53 (1.01)	0.53 (1.09)	0.986
Item 15	0.51 (1.01)	0.44 (0.91)	0.39 (0.87)	0.782
Social domain (Items 13, 14, 15)	1.38 (2.56)	1.23 (2.49)	1.13 (2.21)	0.816
Leisure domain (Items 1, 2, 3, 7, 8, 9)	3.73 (4.97)	3.30 (4.31)	3.28 (4.64)	0.983
Emotional domain (Items 4, 5, 6, 10, 11, 12)	5.48 (5.27)	5.81 (5.64)	4.77 (4.86)	0.285
All together	0.71 (0.75)	0.69 (0.70)	0.61 (0.69)	0.554

Overall no. of patients: 153; males, 47; females, 75; gender unknown in 32 cases. Student's t‐test revealed no significant differences in single items or specific domains of FamilyPso.

## 4 DISCUSSION

Psoriasis not only impairs the patient's health‐related quality of life but also the patient's social lives, ultimately causing a vicious cycle of mutual further impairments of life quality.^11^, ^19^ This has been observed in several studies that closely associate the emotional burden to a negative impact on interpersonal relations, and even marriage can be influenced by psoriasis.^20^ Therefore, the partner's or relative's burden must be reliably assessed and factors worsening it must be identified to be able to offer an effective holistic treatment approach in psoriasis patients. The recently introduced FamilyPso questionnaire provides a solid tool that can be used to measure the disease‐related burden in partners or relatives of psoriasis patients.^21^, ^22^ The importance of this field is also reflected in a prospective study using tildrakizumab to assess the impact of biologic treatment on the well‐being of psoriasis patients and the burden placed on partners and relatives by psoriasis patients.^22^

Overall in this study, we observed that 78.7% of male and 77.3% of female partners reported experiencing a small to an extremely high disease burden from their close relatives and partners, as measured by PFIS (Figure 4), whereas 53.3% of the psoriasis patients reported no impact on their health‐related quality of live (DLQI 0–1) at the same time. Remarkably, a moderate to an extremely large disease burden was observed in 42.5% of the male and 42.7% of the female partners, whereas the impairment of DLQI in the categories ranging from moderate to extremely large in male and female patients was 26.1% and 27.2%, respectively. The emotional burden reached the highest score in our study (Table 5). Furthermore item 4 and item 10, both of which belong to the emotional domain, were among the items scored highest by partners and relatives in this psoriasis cohort (Figure 3 and Table 5). Single‐item and domain analysis results revealed no significant differences regarding the gender of the partners or relatives (Table 5, Table 6). No significant differences were observed with regard to the psoriasis‐related burden (as measured with FamilyPso) in relatives and partners regarding the involvement of certain body areas (i.e. scalp, nails, palms/soles or inverse/genital areas), arthritis, depression or systemic treatment. Adjusting the data used for the PFIS statistical analysis (as was done in a previous study for DLQI) by excluding the items with the answer “does not apply to me” (as was previously done for DLQI)^23^ did not significantly change the study results (data not shown).

**TABLE 6:** FamilyPso items.

Outcome	Item 1	Item 2	Item 3	Item 4	Item 5	Item 6	Item 7	Item 8	Item 9	Item 10	Item 11	Item 12	Item 13	Item 14	Item 15
Not true	60.93%	78.67%	64.00%	14.57%	64.00%	58.00%	49.34%	56.21%	37.09%	39.74%	66.45%	60.81%	63.33%	65.33%	68.87%
Somewhat true	13.25%	6.67%	14.67%	25.17%	12.00%	16.00%	17.11%	11.11%	17.88%	19.21%	13.16%	10.14%	14.67%	12.00%	13.91%
Moderately true	7.95%	2.00%	6.67%	13.25%	6.00%	10.00%	9.21%	5.23%	11.26%	9.27%	5.26%	6.08%	6.00%	2.67%	4.64%
Quite true	4.64%	2.67%	4.00%	22.52%	6.00%	8.67%	5.26%	3.27%	11.26%	11.26%	3.29%	4.05%	2.67%	8.00%	3.97%
Very true	4.64%	0.67%	2.00%	23.18%	4.67%	0.67%	8.55%	3.92%	9.93%	13.91%	3.95%	2.03%	0.67%	3.33%	3.31%
Does not apply	8.61%	9.33%	8.67%	1.32%	7.33%	6.67%	10.53%	20.26%	12.58%	6.62%	7.89%	16.89%	12.67%	8.67%	5.30%

## 5 LIMITATIONS

The cross‐sectional design of this study aside, the low proportion of patients with PASI values > 3 or >10 pose a limitation to this study, as the sizes of these groups limited the power of the statistical analyses. The categorization of PFIS (similar to DLQI) yet needs to be validated in future studies. Moreover, a general limitation of the study is the source of the data from a registry. In registries such as PsoRA data from patients with moderate to severe psoriasis (treated with systemic drugs) predominate and thus do not allow extrapolations to patients with less severe manifestation of the disease.

## 6 CONCLUSION

The results of this cross‐sectional study indicate that the partners and close relatives of 2 out of 5 treated, and well responding psoriasis patients (with a mean PASI <2) still have a moderate to extreme disease burden (as measured by categorized PFIS), whereas only 1 out of 4 patients report moderate to extreme impairments in their quality of life, suggesting that the disease burden of relatives and partners is due to the susceptibility to psoriasis in the patients per se.

## CONFLICT OF INTEREST STATEMENT

Peter Wolf has received research grants from AbbVie, Amgen, Almirall, Astropharma, Boehringer‐Ingelheim, BMS, Celgene, Eli Lilly, Janssen‐Cilag, Leo Pharma, Novartis, Merck Sharp & Dohme, Sandoz, UCB Pharma and Pfizer; received payment or honoraria from Eli Lilly, Celgene; received travel grants from AbbVie; and has participated in boards at Amgen, BMS, Boehringer‐Ingelheim, Celgene, Janssen‐Cilag, Leo Pharma, Eli Lilly, Novartis and UCB.

Wolfgang Weger has received speaker and/or consulting honoraria and/or travel refunds from AbbVie, Amgen GmbHm Almirall, Celgene, Eli Lilly, Janssen, Leo Pharma, Novartis, Merck Sharp & Dohme, Sandoz and Pfizer.

Thomas Graier has received travel grants from Novartis, UCB and Amgen, speaker honoraria from Amgen, Eli‐Lilly and Novartis, and consulting honoraria from Almirall.

Katja Grossschaedl has received grants from Almirall.

Wolfgang Salmhofer reports grants and personal fees from Janssen Amgen, Lilly, AbbVie, and personal fees from Celgene, Leo, and Novartis.

Ulrich Mrowietz has been an advisor and/or received speakers' honoraria and/or received grants and/or participated in clinical trials of the following companies: AbbVie, Aditxt, Almirall, Amgen, Aristea, Biogen, Boehringer‐Ingelheim, Bristol‐Myers Squibb, Celgene, Dr. Reddy's, Eli Lilly, Formycon, Immunic, Janssen‐Cilag, LEO Pharma, Merck, Sharp & Dohme, MetrioPharm, Novartis, Phi‐Stone, Sanofi‐Aventis, UCB Pharma, UNION therapeutics.

## AUTHOR CONTRIBUTIONS

**Katja Grossschaedl**: Conceptualization (equal); Data curation (lead); Formal analysis (equal); Investigation (lead); Methodology (supporting); Project administration (lead); Visualization (equal); Writing – original draft (lead). **Wolfgang Weger**: Conceptualization (equal); Data curation (equal); Investigation (equal); Methodology (equal); Project administration (equal); Supervision (equal); Writing – review & editing (equal). **Thomas Graier**: Formal analysis (equal); Software (equal); Validation (lead); Visualization (lead); Writing – review & editing (equal). **Wolfgang Salmhofer**: Data curation (equal); Project administration (equal); Validation (equal); Writing – review & editing (equal). **Ulrich Mrowietz**: Methodology (equal); Project administration (equal); Supervision (equal); Validation (equal); Writing – review & editing (equal). **Peter Wolf**: Conceptualization (lead); Data curation (equal); Formal analysis (equal); Methodology (lead); Project administration (equal); Resources (lead); Supervision (lead); Visualization (equal); Writing – review & editing (lead).

## ETHICS STATEMENT

This study was conducted in accordance with the Declaration of Helsinki and with the approval of the Medical University of Graz Ethics Committee (Ethic no. 33‐413 ex 20/21).

## Supporting information

Supplementary Material

## Data Availability

The data underlying this article will be shared on request to the corresponding author.
