# Supplementary material for: Psoriasis and its impact on close relatives and partners of patients – A cross‐sectional questionnaire study
Source: Skin Health Dis. 2024 Mar 15;4(3):e355. doi: 10.1002/ski2.355 (PMC11150751; doi:10.1002/ski2.355)
Supplement: Supplementary file 1 — Supplementary Material [file SKI2-4-e355-s001.docx]

**Supplementary tables**

**Table S1: Severity of DLQI**

| DLQI category of impairment, points | No. of males (%) | No. of females (%) | *p*-value comparing males vs. females |
| --- | --- | --- | --- |
| None, 0–1 | 71 (53.0) | 50 (54.3) | *p* = 0.470 |
| Small, 2–5 | 28 (20.9) | 17 (18.5) |  |
| Moderate, 6–10 | 14 (10.4) | 15 (16.3) |  |
| Large, 11–20 | 15 (11.2) | 9 (9.8) |  |
| Extreme, 21–30 | 6 (4.5) | 1 (1.1) |  |

DLQI data are available for a total of 226/250 (90.4%) patients.

**Table S2: Categories of burden of close relatives and partners by Patient Family Impact Score (PFIS)**

| Category of burden, points | No. of males, total *n* = 47 (%) | No. of females, total *n* = 75 (%) | *p*-value comparing males vs. females |
| --- | --- | --- | --- |
| None, 0–1 | 10 (21.3) | 17 (22.7) | *p* = 0.228 |
| Small, ≥ 2–< 9 | 17 (36.2) | 26 (34.7) |  |
| Moderate, ≥ 9–-< 19 | 8 (17.0) | 22 (29.3) |  |
| Large, ≥ 19–< 28 | 9 (19.1) | 5 (6.7) |  |
| Extreme, ≥ 28 | 3 (6.4) | 5 (6.7) |  |

FamilyPso questionnaires were returned by 153 relatives and partners of psoriasis patients. Their gender was unknown in 31 cases; therefore, these data were excluded from this analysis.
